# Gender variation in self-reported likelihood of HIV infection in comparison with HIV test results in rural and urban Nigeria

**DOI:** 10.1186/1742-6405-8-44

**Published:** 2011-12-21

**Authors:** Adeniyi F Fagbamigbe, Joshua O Akinyemi, Babatunde O Adedokun, Elijah A Bamgboye

**Affiliations:** 1 Department of Epidemiology and Biostatistics, Faculty of Public Health, College of Medicine, University of Ibadan, Ibadan, Nigeria

**Keywords:** Urban, rural, sero-positive, HIV/AIDS, validity, behaviour change, Nigeria

## Abstract

**Background:**

Behaviour change which is highly influenced by risk perception is a major challenge that HIV prevention efforts need to confront. In this study, we examined the validity of self-reported likelihood of HIV infection among rural and urban reproductive age group Nigerians.

**Methods:**

This is a cross-sectional study of a nationally representative sample of Nigerians. We investigated the concordance between self-reported likelihood of HIV and actual results of HIV test. Multivariate logistic regression analysis was used to assess whether selected respondents' characteristics affect the validity of self-reports.

**Results:**

The HIV prevalence in the urban population was 3.8% (3.1% among males and 4.6% among females) and 3.5% in the rural areas (3.4% among males and 3.7% among females). Almost all the respondents who claimed they have high chances of being infected with HIV actually tested negative (91.6% in urban and 97.9% in rural areas). In contrast, only 8.5% in urban areas and 2.1% in rural areas, of those who claimed high chances of been HIV infected were actually HIV positive. About 2.9% and 4.3% from urban and rural areas respectively tested positive although they claimed very low chances of HIV infection. Age, gender, education and residence are factors associated with validity of respondents' self-perceived risk of HIV infection.

**Conclusion:**

Self-perceived HIV risk is poorly sensitive and moderately specific in the prediction of HIV status. There are differences in the validity of self-perceived risk of HIV across rural and urban populations.

## Introduction

Effective behaviour change programmes are very important in the effort to reverse the global HIV epidemic. Broad-based behaviour change programmes have played a critical role in reversing the HIV prevalence and incidence in nations with generalized epidemics [1]. One of the several challenges that prevention efforts need to confront is that of perception. Self-perceived risk is a core component of four of the most commonly cited theories used in HIV/AIDS prevention.

These four theories (Health Belief Model, Theory of Reasoned Action, Stages of Change, and AIDS Risk Reduction Model) provide clues on how behaviour changes occur [2]. The health belief model developed in the 1950s is built on the premise that health behaviour is driven by an individual's socio-economic characteristics, knowledge and attitudes with behaviour change hinged on changing individual personal beliefs. The theory of reasoned action proposed in the 1960s is based on the assumption that human beings are usually quite rational and make systematic use of the information available to them [3]. In the 90s, the stages of change model proposed six stages that individuals pass through when changing behaviour: pre-contemplation, contemplation, preparation, action, maintenance and relapse [4]. AIDS risk reduction model developed in 1990 identified three stages involved in reducing the risk of HIV transmission as (1) behaviour labeling, (2) commitment to change, and (3) taking action. Personal risk assessment is a key component of programmes that have used these models. Although these models have played prominent roles in HIV/AIDS interventions, most of them were developed with little focus on gender and interactions of contextual factors. However, intervention programmes designed based on these models focused on perceived risks.

HIV prevention programmes have for long been based on the elements believed to be essential for individuals to initiate and sustain behaviour change. One of these elements is individual's assessment of the risk of being infected. In Nigeria, the HIV and AIDS pandemic has extended beyond the commonly classified high-risk groups (sex workers, men having sex with men, injecting drug users, uniformed service men (Armed forces and Police) and transport workers) and are now common in the general population.

Self-perceived risk is very important in settings like Nigeria where access to HIV testing is limited because behaviour is guided more by perceived risk of infection than unknown HIV status. The Nigeria HIV/AIDS and Reproductive Health Survey 2007 (NARHS) included biomarker for HIV testing [5]; it therefore provides a rich data that can be explored to examine the extent to which perceived risk of infection differs from actual HIV status. This would be the first (to the best of our knowledge) of such national level comparison in the country.

Very few studies have examined self-perceived risk of HIV infection in the country. Many of these were among in-school youths and sex workers [6-8]. Generally, youths perceived themselves to have a low risk of contracting HIV infection (14 - 15%) [6,7], while some female sex workers even incorrectly judged their risk as low [8]. In developed countries, studies among populations at high risk (such as drug users, prostitutes and prisoners) suggests that concurrence between individuals' self-reports of current HIV status and their HIV test results is high for sero-negative people (95-99%) but low for sero-positive people (40-70%) [9-12]. A study of attendees at a voluntary HIV testing centre in Zambia found a 30% rate of incorrect self-reports, with sero-positive patients being only slightly more accurate than sero-negative patients (72% v 60%) [13]. In contrast, a case-control study in Tanzania found no significant difference between perceived risk of infection and HIV status [14]. In addition, a population-based study in Malawi found that only 39% of those whose test was positive reported some likelihood of being infected [15]. From the foregoing, it is obvious that those who have the greatest risk of HIV infection are not likely to rate themselves as having a high chance.

The aim of this study therefore is to compare the self-perceived likelihood of HIV infection to actual HIV test results among rural and urban reproductive age group Nigerians. We also assessed the relationship between respondents' characteristics, and the validity of self-perceived likelihood of HIV infection.

Our analysis was guided by the Health Belief Model which hypothesizes that personal perception is moderated by individual's socio-demographic characteristics (such as age, sex, ethnicity, socio-economic status, knowledge and attitudes. Perceptions are of different components such as perceived susceptibility, perceived threat, perceived benefits and likelihood of action. We focused our analysis on the relationship between socio-demographic characteristics and validity of perceived susceptibility which we hypothesized will vary between rural and urban reproductive age Nigerians.

### Data and Methods

The data used for these analyses was collected by The Nigerian Federal Ministry of Health (FMOH) in collaboration with key partners for the biennial National HIV/AIDS and Reproductive Health Survey Plus (NARHS) in 2007. This was a cross-sectional study among men and women of reproductive age covering all the 36 states and the Federal Capital Territory (FCT) of Nigeria. NARHS Plus was a nationally representative sample of females aged 15-49 years and males aged 15-64 years living in households in rural and urban areas in Nigeria. The NARHS Plus sample was drawn from the updated master sample frame of rural and urban localities developed and maintained by the Nigerian National Population Commission (NPC).

The sampling procedure was a (four-level) multi-stage cluster sampling aimed at selecting eligible persons from households. The first stage was the selection of rural and urban localities while the second stage was the selection of Enumeration Areas (EA) within the selected rural and urban localities. In the third stage, households were systematically selected within the selected EAs. The fourth stage was the actual selection of respondents for interview and HIV testing. Within a state (the administrative division), all eligible persons irrespective of nature of residence (rural or urban) had equal chance of being included in the final sample, hence, the sample selected was self-weighted within state but weighting was required when combined for zonal or national analyses.

Overall, 11,822 respondents were selected for interview of which 11,234 were successfully interviewed resulting in a 5.0% non response rate. This comprised 3,868 (53% male and 47% female) from rural Nigeria and 7,366 (53.4% male and 46.6% female) from urban areas. In all, 53% of the 11,234 respondents are male while 47% are females. Data were collected by personal interview using structured and semi-structured questionnaire. More details on the sampling and fieldwork procedures are available in the survey report [5].

Of the 11,234 that completed the interview, only 9,039 agreed to be tested resulting in 19.5% refusal rate. In the survey, a linked anonymous testing approach with the provision of test results was adopted, whereby individual HIV test results and their respective anonymously completed questionnaires were linked. Further investigation of possible differences between those who accepted HIV test and those that refused was carried out. We found that refusals were evenly distributed by gender and age groups. However, rates of refusals were slightly higher among respondents in North Western Nigeria, those in a union, those with no education, those who had quaranic education and those whose religion was Islam (table not shown).

In this study, we evaluated the validity of self-reported likelihood of HIV infection by comparing the respondents answers to the question "would you rate your chances of getting AIDS (or the virus that causes AIDS) as high, low or no chance at all?" with their HIV antibody test results. The possible answers were "High", "Low", "No risk at all", "Already has AIDS" and "No response". The self-reported likelihood of HIV infection was used as a proxy for HIV status and compared with the HIV antibody test result as the gold standard. For the 'diagnostic test' those who rated themselves as having a high or low chance were categorized as positive while 'no risk at all' was categorized as negative.

### Statistical analysis

Sampling weights were applied in our analysis. The weighting was based on the sampling fractions derived from the sample size and the total population.

Standard epidemiological measures of validity (sensitivity, specificity, negative and positive predictive values) were computed. Sensitivity was computed as the ratio of those who correctly identified themselves as being at risk (i.e. were positive) to all those who were positive on the test. Specificity was calculated as the ratio of those correctly identifying themselves as having no risk to all those who were negative on the test. The positive predictive values were determined by the ratio of true positive to the number of individuals reporting they were at risk of contracting HIV while the negative predictive value was calculated as the ratio of those truly negative to those reporting they were at no risk of HIV. Bivariate analyses were done using the chi square test to investigate association between selected background characteristics and self perceived risk of HIV infection. Finally, we used multivariate logistic regression analysis to assess whether selected respondents' characteristics affect the validity of self-reports. Odds Ratios and 95% confidence intervals were calculated. Analysis was done using SPSS version 15.0. P-values less than 0.05 were considered statistically significant.

### Ethical considerations

The survey was approved by the Nigerian National Health Research Ethics Committee (NHREC). Informed consent was sought from all eligible women and men for their blood to be tested and for further use of the blood sample if necessary. In the case of never-married adolescents' aged 15-17 years, consent was sought from a parent before the adolescent was asked for his/her assent. When there was no parent living in the household, consent was requested from the adult who was in charge of the youth's health and welfare at the time of the NARHS Plus visit and who makes decisions on his/her behalf. The testing approach involved the collection of five blood spots from a finger prick on the same filter paper card and stored as dried blood spots (DBS). A unique random identification number (bar code) was assigned to each DBS and labels containing that code affixed to the filter paper card, the questionnaire, and a field tracking form.

## Results

The HIV prevalence in the urban population was 3.8% (3.0% among males and 4.6% among females) and 3.5% in the rural areas (3.4% among males and 3.6% among females). See Table 1. About six out of every ten respondents (64.5%) from urban Nigeria and 61.7% from rural locations reported that they had no risk at all of being infected with HIV. A very small proportion of respondents from rural (2.2%) and urban (2.3%) areas believed that they had high chances of HIV infection. In the rural areas, females were more likely than males to report a high likelihood of having HIV but the reverse is the case in the urban areas.

**Table 1:** Socio-Demographic characteristics of the respondents

Location	Urban			Rural		
**Characteristics**	**Men** **n(%)**	**Women** **n (%)**	**Both Sexes** **n (%)**	**Men** **n(%)**	**Women** **n (%)**	**Both Sexes** **n (%)**

**HIV status from Test**						

HIV +	51(3.0)	70(4.6)	121(3.8)	107(3.4)	96(3.6)	203(3.5)

HIV _	1627(97.0)	1449(95.4)	3076(96.2)	3063(96.6)	2576(96.4)	5639(96.5)

**Zone**						

North West	253(15.1)	248(16.4)	501(15.7)	868(27.4)	651(24.4)	1519(26.0)

North East	187(11.2)	152(10.1)	339(10.7)	531(16.8)	449(16.8)	980(16.8)

North Central	185(11.1)	161(10.6)	346(10.9)	501(15.8)	443(16.6)	944(16.2)

South West	600(35.9)	598(39.6)	1198(37.6)	457(14.4)	337(12.6)	794(13.6)

South East	222(13.3)	174(11.5)	396(12.4)	305(9.6)	328(12.3)	633(10.8)

South-South	223(13.4)	179(11.8)	402(12.6)	508(16.0)	464(17.4)	972(16.6)

**Education**						

Koranic only	61(3.6)	67(4.4)	128(4.0)	402(12.7)	215(8.0)	617(10.6)

Primary	299(17.8)	315(20.8)	614(19.2)	654(20.6)	573(21.4)	1227(21.0)

Secondary	866(51.7)	720(47.4)	1586(49.6)	1300(41.0)	831(31.1)	2131(36.5)

Higher	371(22.1)	236(15.5)	607(19.0)	260(8.2)	107(4.0)	367(6.3)

None	81(4.8)	180(11.9)	261(8.2)	553(17.5)	947(35.4)	1500(25.7)

**Self -reported HIV perception**						

High	35(2.2)	31(2.1)	66(2.2)	63(2.1)	60(2.6)	123(2.3)

Low	554(34.5)	454(31.5)	1008(33.0)	1099(37.4)	777(33.3)	1876(35.6)

No risk at all	1012(62.9)	958(66.4)	1970(64.5)	1763(5.9)	1487(63.7)	3250(61.7)

Already has HIV	7(0.4)	0(0.0)	7(0.3)	10(0.3)	10(0.4)	20(0.4)

**Age group**						

15-19	369(22.0)	333(21.9)	702(22.0)	696(22.0)	582(21.8)	1278(21.9)

20-24	329(19.6)	299(19.7)	628(19.6)	533(16.8)	542(20.3)	1075(18.4)

25-29	248(14.8)	285(18.8)	533(16.7)	477(15.0)	463(17.3)	940(16.1)

30-39	333(19.8)	386(25.4)	719(22.5)	589(18.6)	621(23.2)	1210(20.7)

40-49	218(13.0)	216(14.2)	434(13.6)	437(13.8)	465(17.4)	902(15.4)

50-64	181(10.8)	0(0.0)	181(5.7)	438(13.8)	0(0.0)	438(7.5)

In Table 2, we compared the self-perceived risk of HIV infection among the respondents with their HIV test result. Almost all the respondents who claimed they have high chances of being infected with HIV actually tested negative (90.9% in urban and 97.6% in rural areas). Similarly, 95.8% of urban dwellers (97.0% male and 94.6% female) and 96.9% in the rural areas (96.9% and 96.9% among men and women respectively) who believed there is no way they could be infected were indeed HIV negative. In contrast only 9.1% in urban areas and 2.4% in rural areas, of those who claimed high chances of been HIV infected were actually HIV positive. On the other hand, against 2.8% (in urban) and 4.3% in rural) of those who claimed very low chances of HIV infection were positive.

**Table 2:** Self-perceived risk of HIV Infection by actual HIV status, NARHS Plus 2007, FMOH, Nigeria.

Location	Urban			Rural		
**HIV perception Versus HIV test**	**HIV+** **n(%)**	**HIV-** **n(%)**	**Total** **n (%)**	**HIV+** **n(%)**	**HIV-** **n(%)**	**Total** **n (%)**

**Men**						

High	2(5.7)	33(94.3)	35	2(3.2)	61(96.8)	63

Low	17(3.1)	537(96.9)	554	40(3.6)	1059(96.4)	1099

No risk at all	30(3.0)	982(97.0)	1012	55(3.1)	1707(96.9)	1762

**Subtotal**	49(3.1)	1552(96.9)	1601	97(3.3)	2827(96.7)	2924

**+p-value**	0.650			0.752		

**Women**						

High	4(12.9)	27(87.1)	31	1(1.6)	60(98.4)	61

Low	11(2.4)	443(97.6)	454	39(5.0)	737(95.0)	776

No risk at all	52(5.4)	906(94.6)	958	46(3.1)	1440(96.9)	1486

**Subtotal**	67(4.6)	1376(95.4)	1443	86(3.7)	2237(96.3)	2323

**+p-value**	0.004*			0.048*		

**Both**						

High	6(9.1)	60(90.9)	66	3(2.4)	120(97.6)	123

Low	28(2.8)	980(97.2)	1008	80(4.3)	1796(95.7)	1876

No risk at all	82(4.2)	1888(95.8)	1970	102(3.1)	3148(96.9)	3250

**Subtotal**	116(3.8)	2958(96.2)	3044	185(3.5)	5064(96.5)	5249

**+p-value**	0.013*			0.088		

*significance at 95%; +p-values from *x*^2 ^test

In urban area, respondents who answered high chances to the question 'Rate your chances of HIV infection' were more likely to test HIV positive than those who answered 'Low chances' or 'No risk at all' (9.1%, 2.8% and 4.2% respectively). In rural areas, those who answered 'Low chances' to the question about their self HIV-assessment were more likely to test HIV positive than others (2.4%, 4.3%, and 3.1% respectively). Among the males, the relationship between self-perceived risk of HIV and HIV positivity was not significant in the urban and rural areas (p > 0.05) but it was significant among females in the urban (p = 0.004) and rural (p = 0.048) settings. For both sexes combined, the relationship was only significant among the urban population (p = 0.013) Table 2.

The result on validity of self reported likelihood of HIV infection showed that 62.1% of respondents were accurate in their HIV self-perceived risk at the time of this survey. Majority (94.2%) of inaccurate self reports were due to respondents' overestimating their risk of being infected, though higher among rural dwellers than urban inhabitants (95% to 92.7%). Among those who tested negative, 63% (64.5% in urban areas and 62.2% in rural areas) had earlier stated they can't be HIV carriers during the time of the survey (specificity) while only 38.6% (28.7% in the urban and 44.8% among rural dwellers) of those who tested positive to HIV test during the survey had stated some chances of being currently infected (sensitivity) (Table 3).

**Table 3:** Accuracy of self-perceived risk of HIV infection by gender and location.

Location	Urban			Rural		
**Characteristics**	**Men** **n(%)**	**Women** **n (%)**	**Both Sexes** **(n)**	**Men** **(n)**	**Women** **(n)**	**Both Sexes** **(n)**

**Accurate Self Reports**						

**TN**	982	906	1888	1707	1440	3147

**TP**	18	15	33	42	40	82

**TN + TP**	**1000**	**921**	**1921**	**1749**	**1480**	**3229**

**Inaccurate Self Reports**						

**FN**	30	52	82	55	46	101

**FP**	570	470	1040	1119	797	1916

**FN + FP**	**600**	**522**	**1122**	**1174**	**843**	**2017**

*Total*	*1600*	*1443*	*3043*	*2923*	*2323*	*5246*

**Accurate Self Reports (%)**	62.5	63.8	63.1	59.8	63.7	61.6

** Inaccurate self reports (%)	95	90	92.7	95.3	94.5	95

**Sensitivity (%)**	37.5	22.4	28.7	43.3	46.5	44.8

**Specificity (%)**	63.3	65.8	64.5	60.4	64.4	62.2

**Negative Predictive Value (%)**	97	94.6	95.8	96.9	96.9	96.9

**Positive Predictive Value (%)**	3.1	3.1	3.1	3.6	4.8	4.1

**Inaccurate Self Reports due to overestimating one's risk. TN = True Negatives, TP = True Positives, FN = False Negatives FP = False Positives

The accuracy of self-perceived risk of HIV were quite close in urban and rural areas (63.1% and 61.6%) but slightly higher among women than men (63.8% vs 60.8%), this is due to overestimation of risk among men.. Self-perceived risk assessments tend to have a clearly higher sensitivity but a lower specificity among the rural dwellers (44.8% and 62.2% respectively) than the urban dwellers (28.7% and 64.5% respectively) (Table 3).

Table 4 (left hand side) shows the results of logistic regression analysis of characteristics that could possibly influence false-positive and false-negative responses. For men, location, geo-political zone and age are significant covariates. The odds of false positive self-perceived risk is much higher in North East zone compared to the North West (OR = 2.38 95% CI 1.92 - 2.93). Similar patterns were observed in the other four zones. Among women, the only significant covariates were geo-political zone, age and education. In the North East zone, women were about 5 times more likely to make false positive assessment than their counterparts from North West zone (95% CI: 4.06 - 6.54). Women between age of 20 and 29 are 1.5 times more likely to give a wrong chance of being infected with HIV. Women with Quranic education are 1.5 times more likely than those without formal education to make false positive assessment about their risk of HIV infection.

**Table 4:** Multivariate logistic regression analysis of false-positive and false-negative responses according to by gender and selected background characteristics

	False-positive				False-negative			
	**Men**		**Women**		**Men**		**Women**	

Variable	AOR	95% CI	AOR	95% CI	AOR	95% CI	AOR	95% CI

**Location**								

Rural	1.15	1.01 - 1.32*	0.95	0.80 - 1.14	0.87	0.53 - 1.41	0.63	0.40 - 0.99

Urban	1.00	(Reference)	1.00	(Reference)	1.00	(Reference)	1.00	(Reference)

**Zone**								

North West	2.38	1.92 - 2.93*	5.16	4.06 - 6.54*	0.50	0.21 - 1.21	0.55	0.22 - 1.36

North East	1.77	1.43 - 2.19*	1.53	1.19 - 1.97*	0.94	0.47 - 1.87	1.28	0.65 - 2.53

North Central	1.57	1.43 - 2.20*	1.44	1.09 - 1.89*	0.96	0.47 - 1.93	0.79	0.39 - 1.61

South West	1.66	1.25 - 2.21*	2.23	1.62 - 3.07*	0.50	0.18 - 1.38	0.27	0.09 - 0.79*

South East	1.50	1.16-1.95*	2.11	1.56 - 2.85*	0.97	0.46 - 2.01	0.60	0.27 - 1.33

South South	1.00	(Reference)	1.00	(Reference)	1.00	(Reference)	1.00	(Reference)

**Age**								

15-19	0.97	0.74 - 1.28	1.22	0.94 - 1.58	0.68	0.24 - 1.95	0.25	0.10 - 0.63 *

20-24	1.41	1.08 - 1.84*	1.25	0.98 - 1.58	0.61	0.21 - 1.79	0.84	0.42 - 1.68

25-29	1.35	1.05 - 1.74*	1.51	1.19 - 1.91*	1.19	0.47 - 2.99	0.74	0.37 - 1.51

30-39	1.45	1.16 - 1.82*	1.10	0.88 - 1.38	1.69	0.76 - 3.73	0.87	0.46 - 1.66

40-49	1.35	1.06 - 1.72 *	-----	--------	1.43	0.61 - 3.36	-----	---------

50-64	1.00	(Reference)	1.00	(Reference)	1.00	(Reference)	1.00	(Reference)

**Marital Status**								

Not living with a sexual partner	0.93	0.78 - 1.12	0.95	0.80 - 1.14	0.93	0.50 - 1.73	1.13	0.66 - 1.93

Living with a sexual partner	1.00	(Reference)	1.00	(Reference)	1.00	(Reference)	1.00	(Reference)

**Religion**								

Islam	1.25	0.62 - 2.50	1.82	0.50 - 6.61	-----	--------	-----	--------

Protestants	1.66	0.83 - 3.30	1.76	0.49 - 6.40	-----	--------	-----	--------

Catholic	1.72	0.86 - 4.46	1.70	0.46 - 6.25	-----	--------	-----	--------

Traditional	1.00	(Reference)	1.00	(Reference)	1.00	(Reference)	1.00	(Reference)

**Education**								

Koranic only	1.27	0.97 - 1.66	1.52	1.06 - 2.18*	1.10	0.42 - 2.89	1.75	0.53 - 5.75

Primary	1.28	0.99 - 1.67	1.65	1.15 - 2.36*	0.82	0.35 - 1.96	3.6	1.66 - 7.81*

Secondary	1.32	0.99 - 1.78	2.17	1.42 - 3.27*	1.48	0.69 - 3.19	4.49	2.05 - 9.80*

Higher	0.91	0.69 - 1.21	1.65	1.18 - 2.30*	1.09	0.43 - 2.75	3.21	1.22 - 8.47*

None	1.00	(Reference)	1.00	(Reference)	1.00	(Reference)	1.00	(Reference)

*Significance at 95%, AOR: Adjusted Odd Ratios; 95% CI: 95% Confidence Interval around the AOR

Also, in the multivariate logistic regression analysis comparing the chances of false-negative responses relative to true-negatives as shown in the right hand side of Table 4, for women the only significant covariates are location, age and education. Women in the rural areas were less likely to underestimate their chances of HIV infection than their counterparts in the urban area (OR = 0.63 95% CI 0.40 - 0.99). Women with secondary education were 3.6 times likely to underestimate their risk (OR = 3.60 95% CI 1.66 - 7.81).

## Discussions and Conclusions

This study has shown that the accuracy of self-perceived risk of HIV were quite close in urban and rural areas and also that self-perceived risk of HIV is poorly sensitive and moderately specific in the prediction of HIV status. There were similarities between males and females and for both gender in rural and urban areas in terms of the accuracy and predictive values. However, there are differences in the sensitivity and false positive rates.

The proportion that reported no chance of contracting HIV in this study is similar to that found in rural Malawi [15]. It is however higher than that reported in a study among pregnant women [16] and among young people 15-24 years [17]. A higher proportion of urban dwellers in our study reported no chance of contracting HIV. This appears counterintuitive as it would have been expected that the higher level of education and exposure in urban areas should translate to a higher knowledge about HIV and thus a higher self-perceived risk of HIV.

HIV/AIDS risk perception, desirability and actual HIV testing have been reported in previous Nigeria National HIV and AIDS and Reproductive Health Surveys (NARHS) in 2003, 2005, and 2007 [5]. The percentage of respondents that said they had low to high chance of being HIV infected were 24.8%, 30.3% and 36.7% for 2003, 2005 and 2007 respectively. The perception varied across gender, location (rural versus urban), educational attainment and age group. In the same vein, although, the proportion that has ever had an HIV test was very low, it increased slightly from 6.8% in 2003 to 14.6% in 2007 [5].

A high false positive (persons rating themselves as having a chance of HIV infection but testing negative to actual HIV screening) and true negative (persons rating themselves as having no chance and testing negative in HIV screening) rates found in this study have also been reported elsewhere [17-19]. It should be noted that these high rates are expected especially from general population surveys of diseases like HIV when the prevalence is relatively low. A setting with low HIV prevalence as low as the rate found in this study has a larger percentage with a negative status thereby increasing the likelihood of true negatives.

The level of accuracy of self-perceived risk (62%) found in this study, although lower than 71% reported in a Malawian study [15], is encouraging as accurate assessment of risk has been shown to be associated with good behaviours such as condom use in last sexual intercourse for example [18]. The slightly higher accuracy among females compared to males found in our study is a reversal of the findings of higher accuracy for males found in Malawi [15]. This difference in accuracy between males and females may be due to a higher overestimation of risk among males. Our study also showed that nearly all (94%) inaccurate self reports were due to overestimation of self-perceived risk, similar proportions have also been reported by other similar studies [15,16].

There are a number of explanations for this very high figure for overestimation of perceived risk of HIV when they are actually HIV negative. It could be a protective mechanism, for example an individual's high risk perception could serve as a reminder to practice safer sexual behaviours even if he/she is HIV negative. On the other hand it could be a reflection of the risky sexual behaviours practiced by respondents which makes them feel at high risk of HIV. A higher perceived HIV risk among those with risky sexual behaviours has been reported [17-19]. Inadequate knowledge about the modes and mechanism of transmission could be another reason for this overestimation. A similar study found that respondents believed that AIDS is highly likely from one act of protected sexual intercourse [17]. This inadequate knowledge may explain the higher false positive rates in rural areas compared to urban areas found in our study.

There were striking differences in the sensitivity of self reported likelihood of HIV between rural and urban areas and also between males and females in urban areas. The reason for a higher chance of correctly reporting HIV risk by rural dwellers could be that these respondents who generally have poorer access to healthcare may expect some benefits such as service provision at the end of the interviews and hence more likely to report a high chance compared to their urban counterparts. The higher sensitivity found among rural respondents indicates that self reports of perceived HIV risk may be of value among those with HIV in these areas particularly in community settings with high HIV prevalence. Urban females may less openly report a higher self-perceived risk even when they are positive because of community norms or expectations in the male dominated Nigeria society where men may more comfortably report sexual behaviours compared to women. The sensitivity results should however be treated with some caution as there were relatively few respondents who tested positive, the denominators of the sensitivity measure.

The results of the logistic regression of false positivity on variables revealed significantly highly odds among males compared to females, though only about 29% higher. This tendency to overestimate HIV risk among males could be an indication of higher level of risky behaviour or response bias whereby males may more likely disclose that they have been unfaithful if married or that they have had multiple sexual partners if they are single.

The separate regressions of false positivity on variables for males and females gave similar results. Lower education was a significant predictor among females. Those with lower education could have a poorer knowledge about modes of contracting HIV and this may explain the high overestimation of risk. This may also explain the higher odds for rural dwellers compared to those in urban as alluded to earlier in this discussion. The higher false positive results for younger respondents may indicate their higher rate of sexual activity and the chance of risky sexual behaviours compared to the older groups.

The regression of false negatives on variables showed that female urban respondents were more likely than those in rural areas to give such reports. The possibility of benefitting from health care services could explain the lower odds of false negative results among rural dwellers. A surprising finding from the logistic regression analysis is the lack of significance of marital status. The perceptions of women concerning their modes of getting the HIV infection will need to be explored to understand reasons for similar false positive rates between marital categories in Nigeria. A limitation of the results of the logistic regression analysis however is in the very high false positive and low false negative results which reduce the power of the test to detect differences.

In the context of the health belief model, we found that age, gender, education and residence have some influence on people's self-perceived risk of HIV infection. One component of the model which we did not include in our analysis is the knowledge of HIV/AIDS among the respondents. The published report of the NARHS survey however demonstrated that there is a sharp difference in knowledge between rural and urban dwellers [5]. This may have contributed to the rural-urban variation in the HIV self-perceived risk.

In conclusion our study has shown important differences in the validity of self-perceived risk of HIV between rural and urban locations. The higher sensitivity of self-perceived risk among rural dwellers especially in those communities with high HIV prevalence make self-perceived risk a potential tool for assessment of HIV status. Interventions are however needed to address the overestimates of self-perceived risk in rural communities and underestimates among urban females as this is important to behaviour change communication programmes aimed at HIV prevention.

## Competing interests

The authors declare that they have no competing interests.

## Authors' contributions

AFF and JOA conceived the study. AFF participated in the study design, in the statistical analysis and in writing the result and coordination. JOA participated in drafting the introduction, in the study design and in the statistical analysis. BOA participated in the study design and in writing the result. EAB participated in drafting the introduction. All authors contributed to the discussion, proofread and approved the final manuscript.
